# Functional Capacity as a Correlate of Health‐Related Quality of Life in Patients With Heart Failure: A Cross‐Sectional Study

**DOI:** 10.1111/nhs.70433

**Published:** 2026-10-19

**Authors:** José Arthur Guimarães dos Santos, Suzana de Oliveira Mangueira, Vinicius Batista Santos, Raúl Juárez‐Vela, João Victor Batista Cabral, Mailson Marques de Sousa

**Affiliations:** ^1^ Federal University of Paraíba João Pessoa Brazil; ^2^ Department of Clinical Nursing Federal University of Paraíba João Pessoa Brazil; ^3^ Department of Nursing Federal University of São Paulo São Paulo Brazil; ^4^ Department of Biomedical Sciences and Health Specialties, Faculty of Health Sciences La Rioja University La Rioja Spain; ^5^ Institute of Neurosciences of Castile and León (INCYL), University of Salamanca, Institute of Biomedical Research of Salamanca (IBSAL) Salamanca Spain

**Keywords:** cross‐sectional studies, functional status, heart failure, quality of life, self‐care

## Abstract

This study aimed to analyze the associations among functional capacity, self‐care, and health‐related quality of life in patients with heart failure (HF). A cross‐sectional correlational study was conducted. Functional capacity was measured with the Duke Activity Status Index, self‐care with the European Heart Failure Self‐Care Behaviour Scale, and health‐related quality of life with the Minnesota Living with Heart Failure Questionnaire. Data were analyzed using Spearman's correlation and simple linear regression analyses. Ninety‐six patients with HF participated. The median functional capacity was 18.90 (IQR: 9.95–32.30), the mean self‐care score was 29.60 (SD = 5.68), and the median quality of life was 51.50 (IQR: 40.00–66.00). Functional capacity was strongly and inversely correlated with health‐related quality of life (*ρ* = −0.700, *p* < 0.001) and accounted for 40.0% of the variance in health‐related quality of life scores. In this sample of outpatients with HF, better functional capacity was associated with better health‐related quality of life.

## Summary

- Functional capacity was significantly associated with health‐related quality of life among people with heart failure.
- Functional capacity assessed with the DASI accounted for 40% of the variance in health‐related quality of life.
- These findings underscore the potential value of targeting functional capacity in patient‐centered educational and rehabilitation interventions for people with heart failure in Northeast Brazil.

## 1 Introduction

Heart failure (HF) is considered the common final pathway for the progression and clinical manifestation of several heart diseases and remains one of the leading global causes of mortality, morbidity, and impaired poor health‐related quality of life (HRQoL) (Beghini et al. 2025). It is a clinical syndrome characterized by signs and symptoms resulting from structural and/or functional cardiac abnormalities that impair ventricular filling or ejection, together with elevated natriuretic peptide levels and/or evidence of cardiogenic congestion (Bozkurt et al. 2021).

Globally, HF affects more than 55 million people, with a prevalence ranging from 1% to 3% in the adult population and proportionally higher rates among older adults (Valente et al. 2026). One‐year mortality ranges from 15% to 30%, and HF is the leading cause of hospitalization after 65 years of age (Savarese et al. 2023). The impact of HF on functional capacity is largely related to reduced cardiac output and impaired oxygen delivery to skeletal muscle, leading to myopathy, exercise intolerance, dyspnea, and fatigue, with consequent deterioration in HRQoL (Gómez et al. 2025).

Adherence to self‐care behaviors is central to maintaining functional capacity, preventing unplanned hospitalizations, and improving health outcomes (Aghajanloo et al. 2021). Self‐care is a continuous process of maintaining health through health promotion practices and disease management in both health and illness. When effectively performed, self‐care contributes to greater clinical stability, well‐being, and HRQoL (Riegel, Jaarsma, et al. 2026). In HF, self‐care often remains suboptimal (Niriayo et al. 2024). Poor adherence to self‐care behaviors is influenced by multiple factors, including health beliefs, habits, and social values, as well as personal characteristics such as perceived control, educational level, social support, cognitive status, and socioeconomic conditions (Sedlar et al. 2021; Riegel, Jaarsma, et al. 2026). Suboptimal self‐care is associated with higher hospital readmission rates, mortality, and poorer HRQoL. HRQoL is an independent predictor of mortality and HF hospitalization regardless of functional class or left ventricular ejection fraction (LVEF), and it also serves as an accessible marker for assessing symptom severity and prognosis in this population (Esteban‐Fernández et al. 2023; Johansson et al. 2021).

The literature includes few studies that simultaneously assess functional capacity, self‐care, and HRQoL among individuals with HF in Latin America, particularly in Northeast Brazil, a region marked by health inequities and underrepresented in the global scientific literature (Santos et al. 2026). Previous studies have examined these variables in isolation, limiting our understanding of the interrelationships that shape prognosis and quality of care in this population (Araújo et al. 2025; Campos et al. 2025; Sedlar et al. 2017; Seid 2020). Therefore, examining these relationships jointly is essential to address this gap, advance knowledge in cardiovascular nursing, and inform clinically relevant and contextually appropriate interventions for the Brazilian health system.

## 2 Objective

To analyze the associations among functional capacity, self‐care, and HRQoL in patients with HF.

## 3 Method

### 3.1 Study Design

This was a cross‐sectional analytical study conducted in accordance with the Strengthening the Reporting of Observational Studies in Epidemiology (STROBE) guidelines.

### 3.2 Study Setting

The study was conducted in the cardiology outpatient clinic of a referral hospital for cardiovascular care in the state of Paraíba, Northeast Brazil.

### 3.3 Population and Sample

The study population comprised patients with a clinical diagnosis of chronic HF confirmed in the medical record who were receiving follow‐up care at the cardiology outpatient clinic. Sample size was estimated considering a finite population of 116 patients, a correlation coefficient of *r* = 0.163 between functional capacity and self‐care management reported in a previous study (Zou et al. 2017), a statistical power of 80%, and a significance level of 5%.

Based on these parameters, the minimum required sample was estimated at 85 patients with HF. To account for possible refusals, 10% was added to the minimum sample size. Initially, 112 individuals were invited to participate and assessed for eligibility. Of these, 14 were excluded for not meeting the predefined inclusion criteria, and two declined to participate. Thus, 96 patients were recruited during the data collection period.

### 3.4 Inclusion and Exclusion Criteria

Patients aged 18 years or older with a diagnosis of chronic HF were included. Patients classified as NYHA Class IV, undergoing hemodialysis, or with dementia or a psychiatric disorder documented in the medical record were excluded.

### 3.5 Variables and Data Collection Instruments

An instrument adapted from a previous study conducted in a population with HF was used for sociodemographic and clinical characterization (Sousa et al. 2018). The instrument included the following variables: place of residence (urban or rural area), age, self‐reported race/skin color, religion, marital status, educational attainment, occupational status, family income, presence of a caregiver, HF etiology, duration of outpatient follow‐up, New York Heart Association (NYHA) functional class (Rohde et al. 2018), associated comorbidities, use of a cardiac implantable electronic device (CIED), cardiovascular risk factors (smoking, alcohol use, and physical activity), nutritional follow‐up, LVEF measured by transthoracic echocardiography, and current medications.

Functional capacity was assessed using the Brazilian version of the Duke Activity Status Index (DASI), and NYHA functional class was obtained from the electronic medical record. The DASI is a well‐established instrument for assessing functional capacity, and each activity included in the scale has a specific value corresponding to its metabolic cost, expressed in metabolic equivalents (METs). The total score ranges from 0 to 58.2, with higher scores indicating better functional performance. The instrument has no cutoff point. The Brazilian version has shown adequate validity evidence, with a Cronbach's *α* of 0.93 (Coutinho‐Myrrha et al. 2014).

NYHA functional class is based on patient‐reported symptoms and ranges from I to IV: Class I, no symptoms; Class II, symptoms during usual physical activities, indicating mild limitation; Class III, symptoms during physical activities less intense than usual, indicating marked limitation, although the patient remains comfortable at rest; and Class IV, inability to perform any physical activity without discomfort, with symptoms present even at rest (Rohde et al. 2018).

Self‐care was assessed using the European Heart Failure Self‐Care Behavior Scale (EHFScBS). The EHFScBS consists of 12 items scored on a five‐point Likert scale, with each item ranging from 1, “I completely agree,” to 5, “I completely disagree.” The total score ranges from 12 to 60, with lower scores indicating better self‐care. The version adapted and validated for the Brazilian population showed a Cronbach's *α* of 0.70 (Feijó et al. 2012).

HRQoL was assessed using the Minnesota Living with Heart Failure Questionnaire (MLHFQ). The MLHFQ consists of 21 items covering two dimensions: Physical, related to the degree of dyspnea and fatigue, and emotional, reflecting concerns such as feeling like a burden, loss of self‐control, worry, and depression. Response options range from 0 to 5 on a Likert scale and assess the impact of the disease on patients' lives. The total score ranges from 0 to 105, with higher scores indicating greater negative impact on HRQoL. The Brazilian version showed a Cronbach's *α* coefficient of 0.97 (Carvalho et al. 2009).

### 3.6 Data Collection Procedures

Data were collected between January and October 2025 in a single session through face‐to‐face interviews lasting approximately 40–50 min. Clinical data were confirmed by reviewing the electronic medical record.

### 3.7 Data Analysis

Data were organized and entered into a Microsoft Excel 2019 spreadsheet and then imported into the Statistical Package for the Social Sciences (SPSS), version 22, and jamovi, version 2.6. Categorical variables were described using absolute and relative frequencies. Numerical variables were described using measures of central tendency and dispersion (mean, standard deviation, median, and interquartile range [IQR]).

Data normality was assessed with the Shapiro–Wilk test, which indicated a nonparametric distribution for FC and self‐care and a parametric distribution for HRQoL. Spearman's correlation coefficient was used to examine the relationship among FC, self‐care, and HRQoL. Correlation strength was classified as weak (0.1–0.29), moderate (0.30–0.49), or strong (≥ 0.50) (Cohen 1992). There were no missing data.

Linear regression analysis was conducted to examine the relationship between variables that were statistically significant in the correlation analysis and the outcome variable, HRQoL. Before interpreting the model, the basic assumptions were evaluated by visually inspecting the quantile–quantile (Q–Q) plot. The confidence intervals (CI) for the coefficients (B) were calculated using bootstrap BCa based on 1000 resamplings. The significance level for the statistical tests was set at 5% with a 95% CI.

### 3.8 Ethical Considerations

This study was approved by the Ethics Committee of the Health Sciences Center of the Federal University of Paraiba, in accordance with the ethical principles defined in the Declaration of Helsinki (Approval No. 7.285.460/2024). All participants provided written informed consent after receiving verbal and written information about the study.

## 4 Results

A total of 96 patients with chronic HF participated in this study. In the sociodemographic profile, most participants were men (*n* = 74; 77.10%), with a mean age of 61.27 years (SD = 13.47); most were aged 60 years or older (*n* = 60; 62.50%). Most participants self‐identified as non‐White (*n* = 70; 72.90%). The predominant educational level was up to 8 years of schooling (*n* = 71; 74.00%), with a mean of 5.0 years (SD = 4.28). Regarding marital status and place of residence, most participants had a partner (*n* = 66; 68.80%), and resided in urban areas (*n* = 75; 78.10%). Most were economically inactive (*n* = 53; 55.20%) and had a family income of at least two minimum wages (*n* = 50; 52.10%). In addition, most did not live alone (*n* = 80; 83.30%) and did not have a caregiver (*n* = 80; 83.30%).

Regarding clinical characteristics, ischemic etiology was the most prevalent (*n* = 60; 62.50%). In terms of clinical severity, patients with NYHA Class I predominated (*n* = 47; 49.00%), as did reduced LVEF (*n* = 66; 68.80%). Most patients had up to two HF‐related comorbidities (*n* = 72; 75.00%) and used up to seven medications daily (*n* = 64; 66.70%). Antihypertensives were the most frequently used medication class (*n* = 126; 79.20%). Twenty‐eight patients (29.20%) had a CIED, most commonly an implantable cardioverter‐defibrillator (*n* = 25; 26.00%).

Most patients had received outpatient follow‐up for HF for up to 5 years (*n* = 80; 83.30%). Regarding lifestyle habits and therapeutic management, few received nutritional follow‐up (*n* = 11; 11.50%), whereas most did not engage in physical activity (*n* = 65; 67.70%), did not smoke (*n* = 83; 86.50%), and did not consume alcoholic beverages (*n* = 84; 87.50%).

Table 1 presents the FC, self‐care, and HRQoL scores.

**TABLE 1:** Scores for functional capacity, self‐care, and health‐related quality of life.

Variables	Mean (SD)	Median (IQR)	Minimum	Maximum
Functional capacity	23.50 (±15.70)	18.90 (9.95–32.30)	4.50	58.20
Self‐care	29.60 (± 5.68)	29.00 (26.00–33.00)	18.00	47.00
Physical HRQoL	18.70 (± 11.50)	18.00 (10.00–27.75)	0.00	43.00
Emotional HRQoL	12.70 (±7.40)	12.00 (6.20–19.00)	0.00	25.00
Total HRQoL	51.50 (± 21.80)	51.50 (40.00–66.00)	3.00	96.00

Abbreviations: HRQoL: health‐related quality of life; IQR, interquartile range.

Table 2 presents the results of the correlation analysis between FC, self‐care, and HRQoL. A strong, statistically significant negative correlation was observed between FC (higher scores indicate better FC) and HRQoL (higher scores indicate worse HRQoL) (*ρ* = −0.700; *p* < 0.000); in other words, as functional capacity increases, quality of life decreases (good). No significant association was found between self‐care and the other variables.

**TABLE 2:** Correlation among functional capacity, self‐care, and health‐related quality of life.

Variables	Spearman's correlation coefficient		
	Functional capacity	Self‐care	Health‐related quality of life
Functional capacity	—	—	—
Self‐care	−0.081	—	—
*p*‐value	0.431	—	
Health‐related quality of life	−0.700	0.127	—
*p*‐value	0.000	0.216	—

A simple linear regression model was used to quantify the significant association between the total DASI score (independent variable) and total HRQoL scores (dependent variable). The coefficient of determination indicated that FC explained 40.0% of the variance in the outcome variable (adjusted *R* ^2^ = 0.400). Analysis of the estimated parameters showed that the total DASI score was a statistically significant predictor of the HRQoL score. The standardized coefficient (standardized *β* = −0.637) indicates that higher DASI scores were associated with better HRQoL scores; in other words, the better an individual's FC, the better the HRQoL (Table 3).

**TABLE 3:** Simple linear regression model for health‐related quality of life.

Variable	Estimate	Standardized coefficient	Standard error	*p*	Adjusted *R* ^2^	BCa 95% CI	
						Lower	Upper
Functional capacity	−0.888	−0.637	0.111	< 0.001	0.400	−1.141	−0.673

## 5 Discussion

In this study, FC scores were higher than those reported in studies conducted in the United States (Augusto et al. 2025), indicating better FC in this sample. Self‐care scores were similar to those observed in a Japanese study (Yoshinaga et al. 2022) and higher than those reported in Southeast Brazil (Lima et al. 2023), suggesting worse self‐care. The mean HRQoL score was also higher than that reported in a multicenter study involving Sweden, Italy, Israel, the Netherlands, Germany, and the United States (Westas et al. 2026), indicating poorer HRQoL among participants in the present study.

Self‐care was not associated with the HRQoL. Self‐care is a dynamic and multifaceted process that encompasses pharmacological and nonpharmacological actions essential for maintaining the clinical stability of HF. Several factors can influence this behavior, such as age, previous experience with the disease, cultural preferences, health literacy, skills, cognition, presence of anxiety and depression symptoms, social support, and self‐efficacy (Riegel, Lee, et al. 2026). This finding is particularly relevant, as it shows that the participants' self‐care behavior is not yet consolidated, given the multiplicity of factors that influence it.

The relationship between self‐care and HRQoL in patients with HF is not linear. Previous studies have reported heterogeneous results (Seid et al. 2023). In Iran, a study with a sample of 217 participants found no association between the variables (Tapak et al. 2025). A systematic review of 30 studies showed inconsistencies in the associations between most studies (Sedlar et al. 2017). This finding reinforces the need for targeted and individualized interventions, as generic strategies for self‐care guidance tend to be insufficient given the complexity of this process. In this sense, it is up to nursing and multidisciplinary teams to identify the specific barriers of each individual and develop continuous strategies that favor the consolidation of self‐care over time, with a potential impact on the clinical stability and HRQoL of patients with HF.

The evaluation of FC in patients with HF is a crucial element in determining clinical outcomes, as FC has been identified as a significant predictor of HRQoL. In this study, linear regression analysis corroborated existing literature by confirming FC as a strong predictor of this outcome. Enhancements in physical performance not only improve physiological aspects but also foster greater autonomy in daily activities, thereby directly affecting health satisfaction. Furthermore, FC has been recognized as a key predictor of patient‐reported outcomes, particularly within the physical domain of HRQoL (Wu et al. 2025).

HF impairs physical and functional capacity, with a sustained negative impact on HRQoL. This decline is driven by multiple factors, including depression, fatigue, pain, and insomnia, self‐care that reduce functional performance and necessitate integrated care approaches (Martens et al. 2023; Sedlar et al. 2017). Cardiac rehabilitation plays a central role in this context, as structured exercise training programs promote clinically relevant improvements in HRQoL (Baral et al. 2024). However, adherence and access remain limited, particularly in low‐ and middle‐income countries.

Cardiac rehabilitation should be a comprehensive and progressive intervention designed based on clinical conditions and the patient's experiences, satisfaction, and expectations (Bäck et al. 2026). Nursing is strategically positioned to promote the FC. Clinical trials have demonstrated that nurse‐led interventions based on education, self‐management, and continuous follow‐up improve self‐care and functional status while reducing readmissions and healthcare costs (Chen et al. 2025). Innovative strategies, such as the exergames evaluated in the Swedish HF‐Wii study, have also shown benefits across exercise capacity, fatigue, dyspnea, well‐being, HRQoL, and psychological symptoms (Klompstra et al. 2025). These findings reinforce the potential of nursing to implement accessible, innovative, and sustainable cardiac rehabilitation strategies.

## 6 Study Limitations

The cross‐sectional design and single‐center data collection in Northeast Brazil limit causal inference and the generalizability of the findings. In this study, the majority of the sample was male, in NYHA Class I, and had ischemic etiology, which requires caution in interpreting the findings. In addition, the collection instruments are subject to memory bias and social desirability bias. Multicenter longitudinal studies are needed to examine the relationship between these variables over time.

## 7 Conclusion

A significant correlation was found between functional capacity and HRQoL, indicating that greater functional capacity was associated with better perceived HRQoL. Functional capacity explained 40% of the variance in HRQoL scores, confirming its role as an independent predictor.

## 8 Relevance for Clinical Practice

These findings expand the evidence on the relationships among functional capacity, self‐care, and HRQoL in HF, with direct implications for nursing practice. Recognizing functional capacity as an independent predictor of HRQoL reinforces nurses' central role in systematic assessment and in planning targeted educational and rehabilitation interventions for this population, with potential impact on self‐care adherence and clinical outcomes.

## Author Contributions

**José Arthur Guimarães dos Santos:** investigation, writing – original draft, writing – review and editing, methodology, formal analysis, project administration, data curation, validation, conceptualization. **Suzana de Oliveira Mangueira:** writing – original draft, writing – review and editing, formal analysis, methodology, validation. **Vinicius Batista Santos:** writing – original draft, writing – review and editing, formal analysis, validation, methodology. **Raúl Juárez‐Vela:** writing – original draft, writing – review and editing, validation, methodology, formal analysis. **João Victor Batista Cabral:** writing – original draft, writing – review and editing, validation, methodology, formal analysis. **Mailson Marques de Sousa:** conceptualization, writing – original draft, writing – review and editing, investigation, methodology, validation, formal analysis, project administration, supervision.

## Funding

This study was funded by the Coordenação de Aperfeiçoamento de Pessoal de Nível Superior—Brasil (CAPES) through a master's scholarship (Funding Code 001).

## Consent

All participants provided written informed consent after receiving verbal and written information about the study.

## Conflicts of Interest

The authors declare no conflicts of interest.

## Data Availability

The data that support the findings of this study are available from the corresponding author upon reasonable request.
